# Modulation of Endosome Function, Vesicle Trafficking and Autophagy by Human Herpesviruses

**DOI:** 10.3390/cells10030542

**Published:** 2021-03-04

**Authors:** Eduardo I. Tognarelli, Antonia Reyes, Nicolás Corrales, Leandro J. Carreño, Susan M. Bueno, Alexis M. Kalergis, Pablo A. González

**Affiliations:** 1 Millennium Institute on Immunology and Immunotherapy, Santiago 8330025, Chile; eitognar@uc.cl (E.I.T.); azreyes@uc.cl (A.R.); ncorrales@uc.cl (N.C.); leandrocarreno@uchile.cl (L.J.C.); sbueno@bio.puc.cl (S.M.B.); akalergis@bio.puc.cl (A.M.K.); 2 Departamento de Genética Molecular y Microbiología, Facultad de Ciencias Biológicas, Pontificia Universidad Católica de Chile, Santiago 8331150, Chile; 3 Programa de Inmunología, Instituto de Ciencias Biomédicas, Facultad de Medicina, Universidad de Chile, Santiago 8380453, Chile; 4 Departamento de Endocrinología, Facultad de Medicina, Escuela de Medicina, Pontificia Universidad Católica de Chile, Santiago 8320000, Chile

**Keywords:** human herpesviruses, autophagy, endocytosis, lysosomes, trans-Golgi network, ESCRT, exocytosis, viral

## Abstract

Human herpesviruses are a ubiquitous family of viruses that infect individuals of all ages and are present at a high prevalence worldwide. Herpesviruses are responsible for a broad spectrum of diseases, ranging from skin and mucosal lesions to blindness and life-threatening encephalitis, and some of them, such as Kaposi’s sarcoma-associated herpesvirus (KSHV) and Epstein–Barr virus (EBV), are known to be oncogenic. Furthermore, recent studies suggest that some herpesviruses may be associated with developing neurodegenerative diseases. These viruses can establish lifelong infections in the host and remain in a latent state with periodic reactivations. To achieve infection and yield new infectious viral particles, these viruses require and interact with molecular host determinants for supporting their replication and spread. Important sets of cellular factors involved in the lifecycle of herpesviruses are those participating in intracellular membrane trafficking pathways, as well as autophagic-based organelle recycling processes. These cellular processes are required by these viruses for cell entry and exit steps. Here, we review and discuss recent findings related to how herpesviruses exploit vesicular trafficking and autophagy components by using both host and viral gene products to promote the import and export of infectious viral particles from and to the extracellular environment. Understanding how herpesviruses modulate autophagy, endolysosomal and secretory pathways, as well as other prominent trafficking vesicles within the cell, could enable the engineering of novel antiviral therapies to treat these viruses and counteract their negative health effects.

## 1. Introduction

Cell entry is a critical step for initiating the replication cycle of viruses and for the evasion of host antiviral responses. Similarly, the egress of newly synthesized infectious particles from the infected cell is a determinant step for virus perpetuation and spreading onto other cells and organisms [1,2,3,4]. Because viruses are obligate intracellular pathogens, the manipulation of host cellular molecular determinants and processes is key for their perpetuation [5,6,7,8]. To achieve this, viruses have undergone natural selection over time and co-evolved with their hosts, allowing them to harness fundamental cellular processes in their favor. Among the relevant cellular processes that impact viral infection and yield from infected cells are those related to membrane trafficking, such as endolysosomal and secretory pathways, as well as autophagy [9,10,11]. Membrane trafficking functions allow cells to maintain their homeostasis and also to respond to diverse environmental factors, such as pathogens [12,13]. Although these processes can lead to virus degradation and clearance if undisrupted by these pathogens, once they are harnessed and modulated appropriately by viruses, they can benefit viral infection by favoring viral infection, expansion and dissemination by promoting cell lysis [14,15]. For herpesviruses, several steps of the replication cycle rely on factors involved in the trafficking of membrane components, such as host determinants used for viral genome localization within the cells, viral protein synthesis, as well as lipid envelopment-related processes [16].

At present there are eight known human herpesviruses, classified as follows. Human herpesviruses 1 (HHV-1) and 2 (HHV-2), commonly known as herpes simplex viruses type 1 (HSV-1) and type 2 (HSV-2) are herpesviruses mainly associated with skin and mucosal lesions, blindness and encephalitis [4]. Human herpesvirus 3, better known as varicella-zoster virus (VZV), is the cause of chickenpox and shingles, which manifest as scattered vesicular lesions on the skin and painful dermatome rashes, respectively [17]. Epstein–Barr virus is the causative agent of infectious mononucleosis, nasopharyngeal carcinoma and several lymphoproliferative diseases [18], and it also produces EBV-associated gastric cancer [19]. Human cytomegalovirus (HCMV) typically does not produce noticeable clinical manifestations in healthy individuals; however, in immunocompromised people, it is attributed to promoting the occurrence of pneumonia, gastrointestinal ulcers, hepatitis and retinitis [20]. Additionally, congenital HCMV infection is the most common infection during pregnancy worldwide and increases the probability of acquiring sensorineural hearing loss (SNHL) and the development of intellectual disabilities in children [21,22]. Human herpesvirus 6 (HHV-6) collectively refers to two members of human roseoloviruses, HHV-6A and HHV-6B, which infect individuals early in life and are mostly asymptomatic [23]. These viruses tend to reactivate in immunocompromised individuals with acquired immune deficiency syndrome (AIDS) or transplanted patients, causing complications such as encephalitis [17,24]. HHV-6B is the causative agent of exanthema subitum in young children. Another human roseolovirus is human herpesvirus 7, which rarely elicits clinical manifestations. Nevertheless, it has been commonly grouped with diseases produced by HHV-6 viruses [25]. Lastly, Kaposi’s sarcoma-associated herpesvirus (KSHV, HHV-8) is known for causing sarcoma cancer in immunocompromised individuals, mainly those who have AIDS [26].

Furthermore, as an important trait of human herpesviruses, these viruses have developed numerous mechanisms for persisting in the human body [4]. Indeed, the infection outcome depends on the subversion of numerous cellular processes. In this review, we focus on those disrupted by herpesviruses that are related to membrane trafficking, namely endolysosomal and exosomal vesicle factors, as well as autophagy [27,28]. Given the relevance of these steps in the replication cycle of these viruses, these cellular processes may be attractive targets for prospective therapeutic approaches.

## 2. Endocytosis-Mediated Herpesvirus Entry

### Role of Endocytosis in Herpesvirus Entry

Endocytosis is a membrane trafficking process in which the cell internalizes macromolecules or small particles into the intracellular medium to direct them to different compartments, where they may serve to fulfill a broad range of physiological processes [29]. Endocytosis’ main role is to support nutrient uptake, and it may also promote cell development through the internalization of morphogenic compounds and regulate cell adhesion [30]. There are two main categories of endocytic processes—phagocytosis, which is found mostly in mammalian cells, and pinocytosis, which is present in most cell types. Phagocytosis consists of the uptake of large particles such as cellular debris or pathogens by specialized cells including macrophages, dendritic cells, monocytes and neutrophils [31,32]. On the other hand, pinocytosis consists of the uptake of small particles or soluble compounds. It comprises four types: macropinocytosis, clathrin-mediated endocytosis, caveolae-mediated endocytosis and clathrin- or caveolae-independent endocytosis pathways (GLIC/GEEC or ARF6-dependent) lacking dynamin association [33], which overall provide different degrees of selectivity for the internalized compounds [29,34,35,36,37].

**Figure 1:**
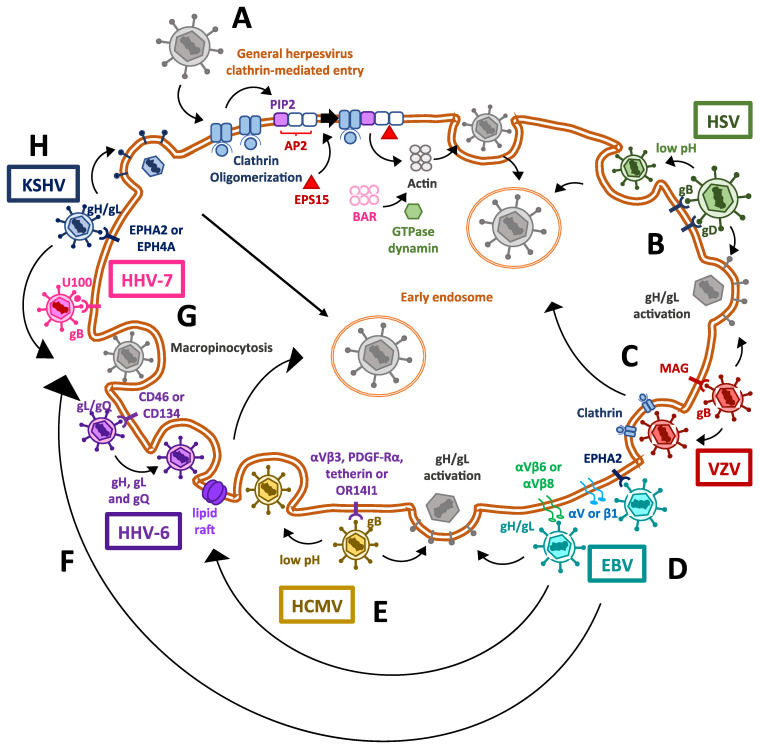
Mechanisms used by human herpesviruses to enter host cells. (**A**) Clathrin-mediated entry for herpesviruses in general. Herpesviruses induce clathrin oligomerization, together with the recruitment of host factors and adapter proteins, which also induce the polimerization of actin filaments and lastly the invagination of the cell membrane, thanks to the GTPase activity of dynamin. (**B**) Cell entry mechanisms exploited by human herpesviruses type 1 and type 2 (HSV-1 and HSV-2). The binding of viral glycoproteins B and D to their ligands triggers dimerization of the gH/gL complex, which causes the activation of the fusogenic activity of gB, enabling the release of the viral capsid into the cytosol. On the other hand, viral capsid delivery through the endosomal pathway has also been described, which is associated with endosomes having a low pH. (**C**) The mechanism used by varicella-zoster virus (VZV) for entering cells is also mediated by the gH/gL complex, but clathrin-mediated entry has also been described for this virus. (**D**) The Epstein–Barr virus (EBV) has been described to enter the cell through its binding to the EPHA2 receptor. Alternatively, other entry pathways exist, such as that mediated by BMRF-2 binding to integrins, causing gH/gL complex activation and gB-mediated fusion of membranes; lipid raft-mediated endocytosis; or viral entry through micro- and micropinocytosis. (**E**) The human cytomegalovirus (HCMV) mainly enters the cell via the activation of the gH/gL complex, triggering gB-mediated membrane fusion, but a low pH-dependent endocytosis mechanism has also been reported, involving a viral protein complex binding to host OR14I1. (**F**) The entry mechanisms exploited by human herpesviruses 6 (HHV-6; HHV-6A and HHV-6B) mainly occurs by endocytosis through lipid rafts. (**G**) The only entry mechanism described so far for the human herpesvirus 7 (HHV-7) is mediated by a fusogenic process mediated by the gH/gL complex. No endocytic pathway has been described yet for this virus. (**H**) Finally, Kaposi’s sarcoma-associated herpesvirus (KSV) infects the cells through a gB-mediated membrane fusion pathway, clathrin-mediated endocytosis and may also induce micro- and macropinocytosis.

Given the ubiquitous ability of cells to perform endocytosis, several viruses utilize endogenous endocytic mechanisms to some extent in order to facilitate the infection of target cells [2]. Human herpesviruses mostly undergo cellular infection processes that involve the fusion of the viral envelope with the plasma membrane of host cells, enabling viral capsids to be internalized into the cytoplasm (Figure 1) [38,39]. The fusion of the lipid bilayer membranes of the virion and the cell requires viral attachment to the cell surface, accomplished by one or more surface viral glycoproteins to specific receptors in the host cell [4]. Alternatively, some human herpesviruses can also enter the cells through endocytosis (Figure 1) [40]. Noteworthy, endocytosis-based viral entry may occur more frequently, preferably over membrane fusion, in some cell types but not in others [41,42].

Herpesvirus internalization mediated by endocytosis can occur due to clathrin-mediated endocytosis, which requires the virus to induce the oligomerization of a clathrin coat in the inner side of the cell membrane [43,44]. Endocytosis then occurs due to the assembly of host protein complexes recruited to the host membrane thanks to protein adaptors, such as the AP2 complex [34]. The AP2 complex attaches to membrane lipids, particularly phosphatidylinositol 4,5-bisphosphate (PIP2), altogether binding to cargo molecules targeted to the clathrin vesicle [45]. This causes clathrin to act as a scaffold protein, together with the epidermal growth factor receptor substrate 15 (EPS15), to fold and interact with the adaptors and themselves and cluster together [46]. Following clathrin clustering, actin filaments polymerize to form a module for subsequent intracellular transit [47]. Ultimately, membrane invagination and scission [48] occur when the actin module recruits the GTPase dynamin, which is involved in membrane constriction, as well as Bin/Amphiphysin/Rvs (BAR)-domain proteins, which are required for producing curvatures in the lipid bilayer [49].

Human herpesviruses 1 and 2 surface glycoproteins have been shown to play a critical role in the attachment of herpesviruses to the cell surface. The glycoprotein B (gB) protein of HSV-1 and HSV-2 is involved in the attachment of the virions to heparan sulfate moieties on the cell surface, whereas glycoprotein C (gC) also participates in this process for HSV-1, but not for HSV-2 [50,51]. On the other hand, the HSV-1 gB protein has also been reported to bind to paired immunoglobulin-like type 2 receptor alpha [52]. After gB binds the corresponding host factors, glycoprotein D (gD) of either HSV-1 or HSV-2 binds non-immune cells primary to nectin cell adhesion molecule-1 (NECTIN1, HVEC) or nectin-2 (NECTIN2, HVEB), or in immune cells to the herpesvirus entry mediator (HVEM, TNFRSF14) [53,54,55,56]. This promotes the activation of the viral gH/gL complex. This gH/gL complex then enables the fusogenic activity of gB, which is a type III fusion protein and which mediates virus and host membrane fusion and capsid entry into the cytoplasm [57,58,59].

However, there is evidence that HSV-1 virions enter preferably through endocytosis in epithelial cells, such as retinal pigment epithelial cells, human epidermal keratinocytes and human conjunctival epithelial cells [41,42]. The preference towards endocytosis-mediated entry over membrane fusion likely occurs once the prefusion form of glycoprotein B (gB) undergoes conformation changes at a low pH, which promotes the fusogenic activity of this viral protein with budding endosomes before the remaining entry-related viral glycoproteins interact with their cell ligands [40,60,61]. Interestingly, it has been recently reported that gC of HSV-1 facilitates conformational changes in gB, which may regulate intracellular viral uptake and lead to the preference of low-pH endosomes for viral entry in human epidermal keratinocytes [62]. Endocytosis and virion delivery into acid endosomal compartments seems to support HSV-2 infection of dendritic cells (DCs) that are present in the site of primary infection, as impairing this cellular pathway decreases the rate of infection of these cells [63].

Although varicella-zoster virus (VZV) entry into target cells is not fully understood, it is presumed to occur similarly to HSV-1 and HSV-2 through a membrane fusion process, in which several envelope glycoproteins participate [64]. To carry on the initial attachment event, gB of VZV has been reported to bind to myelin-associated glycoprotein [65]. Other undetermined viral envelope proteins are also predicted to interact with the mannose-6-phosphate (M6P) receptor [66]. The interaction of gB of VZV with MAG causes gH/gL to act as the core fusogenic complex, promoting viral membrane fusion with the host lipid bilayer [65].

Alternatively, VZV may enter the cell through an endocytosis process mediated by clathrin, in which the tyrosine-based endocytosis motif (YNKI) in the cytoplasmatic tail of gH is required [67]. Interestingly, this sequence has been shown to be the main component responsible for regulating the fusogenic activity of gH and cell-to-cell [68].

Infection with EBV virus seems to have a preferable tropism towards epithelial and B lymphocytes [69]. Recently, ephrin receptor A2 (EPHA2) was identified as a key host factor, required as the fusion receptor for membrane fusion-dependent viral entry into epithelial cells, whereas knocking out integrins did not affect EBV infectivity in HEK293 cells [70]. Previously, it was described in epithelial cells that EBV BMRF-2 protein interacts with αv or β1 integrins to facilitate basolateral infection [71]. After BMRF-2 interaction with the abovementioned integrins, EBV uses the gH/gL complex to interact either with the αvβ6 or αvβ8 integrins as receptors to trigger a conformational change in gB for membrane fusion and virus internalization to occur [72]. Interestingly, EBV has also been reported to use an alternative route for entering nasopharyngeal epithelial cells, which is lipid raft-mediated endocytosis or micropinocytosis [73]. Interestingly, the route used for infecting oral epithelial cells is transcytosis, through apical macropinocytosis, whereas viral exit occurs via basolateral clathrin-mediated endocytosis [71].

On the other hand, Epstein–Barr virus (EBV) infection of B cells involves endocytosis through the fusion of the viral membrane with endosomal membranes [70,74]. The viral glycoprotein gp350 binds to CD21 on the B cell surface as an initial attachment [75] and thereafter glycoprotein gp42 forms a stable complex with the gH/gL complex, promoting them to bind to the human leukocyte antigen class II (HLA-II) and triggering the fusogenic activity of gB with consequent viral and host membranes fusing, allowing the entry of viral components into the cell [76,77].

Human cytomegalovirus (HCMV) entry into host cells bears a resemblance with HSVs in regards to the fusion process, with glycoprotein gB mediating viral attachment to heparan sulfate glycosaminoglycans [78,79]. Nevertheless, HCMV gB has also been shown to have the ability to engage other receptors, such as tetherin [80], platelet-derived growth factor-α receptor (PDGF-Rα) [81] and integrins such as αvβ3 [82,83]. The gH-gL complex also participates in HCMV entry by triggering virus and cell membrane fusion through gB. However, in endothelial and epithelial cells, but not fibroblasts, this complex requires the formation of a pentamer with three other viral proteins, namely, UL128, UL130 and UL131, to be functional [84,85]. Furthermore, it has been shown that HCMV can mediate its entry into epithelial and endothelial cells through the interaction of the viral fusion protein complex with the host receptor OR14I1, followed by a low-pH dependent-endocytosis process [86,87].

The distinct members of the roseoleovirus genus human herpesvirus 6, namely, HHV-6A and HHV-6B, display differences compared to the other herpesviruses detailed above. These viruses use a different configuration of glycoproteins for cell attachment and entry during their lytic cycles [88]. Although they also use gB for attaching to heparan sulfates, gH forms a complex with gL and gQ, which oversees the recognition and attachment of the virus to the ubiquitous receptor molecules CD46 for HHV-6A, and CD134 for HHV-6B [89]. This sequence of molecular interactions then triggers the fusion of the viral and cellular membranes [89,90,91,92,93]. Current evidence suggests that following cell surface binding, HHV-6A and HHV-6B glycoprotein complexes gH-gL-gQ mediate cell entry primarily via endocytosis in T cells (T lymphoblastoid cell line) [94,95], seemingly through lipid rafts [96]. Consequently, it has been reported that endocytosed virions travel through the cytosol, where, by unknown means, the capsids suffer de-envelopment, which grants them access to the external nuclear membrane in the cell [97].

Human herpesvirus 7 uses a similar entry mechanism to HHV-6A and HHV-6B, with the viral proteins gB and U100 both involved in the attachment of the virus to heparan sulfate proteoglycans on the cell surface [98,99], or alternatively to CD4 by means of a yet-unknown viral protein [100]. Notably, an alternative mechanism of entry via endocytosis has not been described yet for HHV-7 [98,99].

Finally, Kaposi’s sarcoma-associated herpesvirus (KSHV) also uses glycoproteins to promote viral entry through clathrin-coated mediated endocytosis. In order to do this, KSHV requires the viral gH/gL complex to bind to the ephrin-binding site of the EPHA2 receptor [101]. Consistent with this notion, an ephrin antagonist applied to epithelial or endothelial cells inhibits viral entry [102]. KSHV may also bind to the EPHA4 receptor in epithelial cells with higher efficiency than the EPHA2 receptor [103]. Notably, KSHV has also been observed to preferentially enter endothelial cells via micropinocytosis [104] by inducing tyrosine phosphorylation of c-Cbl to promote the translocation of virus receptors, such as the integrin αVβ3, into lipid rafts [105]. Furthermore, in early infection events, the adaptor protein c-Cbl interacts with actin and ubiquitinates the myosin light chain IIA, thus enabling bleb formation, which in turn allows for macropinocytosis-mediated viral entry [106].

Overall accumulating evidence reveals multiple entry mechanisms into cells by herpesviruses, with more than one type of entry for single viruses in many cases (Figure 1). Moreover, there is evidence that some herpesviruses can trigger actin remodeling, which causes ruffling of the host plasma membrane and macropinocytosis for endocytic uptake [73,84,107,108]. In these cases, virus-containing vesicles consequently acidify, mature and fuse with late endosomes or lysosomes [39]. Nevertheless, it remains to be elucidated which signals determine that a herpesvirus prefers to use the endocytic pathway for entry or membrane fusion processes. Interestingly, the essential viral molecular determinants participating in membrane fusion and virus entry for some herpesviruses, such as VZV and HHV-7, are still unknown.

## 3. Role of Autophagy during Herpesvirus Infections

### 3.1. Autophagosome Function

Autophagy consists of a cellular process that promotes the elimination of damaged or senescent organelles and proteins to maintain cellular homeostasis and prevent metabolic deregulation [5,27]. Furthermore, autophagy is important in cellular antiviral responses because it can help cells to control virus takeover, promote cell survival, favor antigen presentation, regulate the initiation of inflammatory cytokine responses and target pathogens for degradation [109]. Some of the immune-modulatory aspects of autophagy are accomplished by Toll-like receptors (TLRs), pathogen recognition receptors (PRRs), which recognize pathogen-associated molecular patterns (PAMPs) from the virus that are present in endosomes and belong to the autophagosome pathway, causing signaling cascades with different outcomes [110]. Importantly, autophagy participates in providing the cell with a source of virus-derived antigens that can be loaded onto major histocompatibility molecules (MHC) that are involved in antigen presentation to immune cells [111]. This is achieved by autophagosomes fusing with multivesicular compartments containing MHC class I (MHC-I) molecules that enable antigen presentation to CD8+ T cells [112]. Furthermore, fusion of autophagosomes with MHC class II (MHC-II)-containing compartments allows the infected cell to perform type-2 cross-presentation, in which case intracellular antigens are targeted for presentation to CD4+ T cells [113].

During autophagy, small lipid bilayers that enclose diverse contents in the cytosol are recruited to form an autophagosome [114]. The engulfed contents are then targeted for degradation by delivering them to lysosomes [115]. The sequential steps that mediate the synthesis of autophagosomes relate to a series of enzymatic reactions involving, among others, autophagy-related (ATG) proteins [116]. In mammalian cells, the initiation of autophagosomes begins with crescent-shaped pre-autophagosome buds, termed phagophores, that hail from the endoplasmic reticulum (ER) and require ATG13 to form a complex with ULK1 and ULK2 [115]. Afterwards, nucleation occurs due to ULK1 phosphorylation, which in turn activates Beclin-1, the ATG6 orthologue in mammals that (along with vacuolar protein sorting 15 and PIK3C3/Vps34) forms a membrane-bound complex that acts as a class III phosphatidylinositol-3 kinase (PI3K) [117,118]. This PI3K complex phosphorylates phosphatidylinositol into phosphatidylinositol-3-phosphate (PIP3), which is recruited to the budding autophagosome membrane [119]. This, in turn, leads to an elongation step; as the phagophore becomes larger the complex recruits microtubule-associated light chain 3 (LC3) and other ATG proteins, such as ATG5, ATG12 and ATG16 [120]. Afterwards, LC3 is primed by E1-like enzyme ATG7 for cleavage by protease ATG4 and converts it into LC3-I [121,122]. Finally, autophagosome maturation is achieved by the action of an E2-like enzyme, ATG3, which allows LC3-I lipidation and its transformation into LC3-II through a ubiquitin-like mechanism [123]. LC3-II is the main membrane-bound protein that characterizes mature autophagosomes capable of fusing with lysosomes, with which the content is combined to allow the degradation of organelles and proteins by acidic proteases, such as cathepsins [124,125]. Rab7, SNARE proteins, ATG14 and ATG6 also play roles in autophagosome-lysosome fusion, with the latter also participating to some extent in endosomal membrane trafficking [118,126,127]. Furthermore, autophagosomes can fuse with endosomes to create intermediary membrane structures termed amphisomes, which have been described to form by reactive oxygen species generated by NADPH oxidase-containing endosomes that recruit LC3-containing organelles [128].

Depending on the herpesvirus species, the autophagy pathway may be suppressed in infected cells to promote virus fitness and replication (e.g., HSV-1 and HSV-2), or may be induced to harness its components in order to promote viral replication and yield [27]. Further details on how these viruses modulate autophagy are described below.

### 3.2. Herpesvirus Modulation of Autophagosome Function

**Figure 2:**
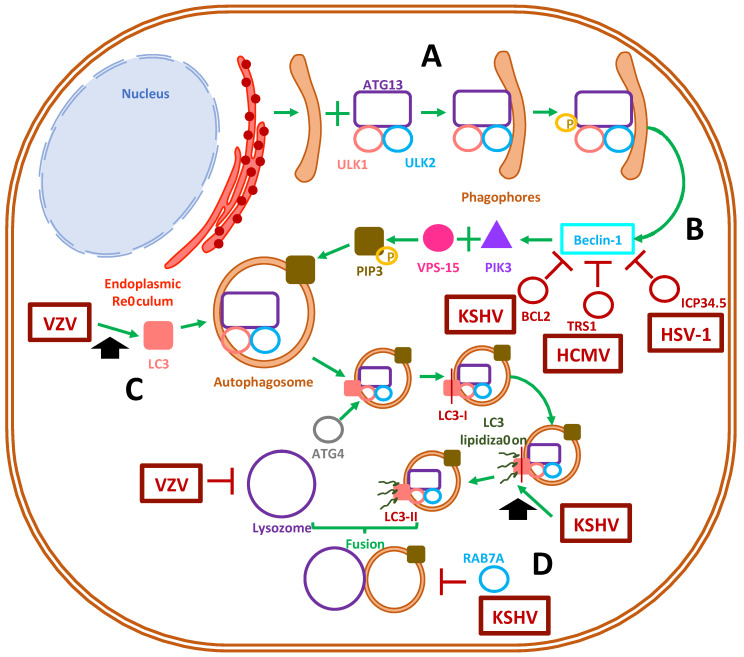
Modulation of autophagosome formation by herpesviruses. (**A**) Autophagosome formation in mammalian cells begins with the materialization of the phagophore, together with the recruitment of host proteins autophagy-related protein 13, ULK1 and ULK2. (**B**) Afterward, ULK1 phosphorylation activates the beclin-1 protein and triggers the recruitment of proteins that finally recruit the class-III phosphatidylinositol-3 kinase to the autophagosome membrane. The activation of beclin-1 is inhibited by the viral proteins BCL2, TRS1 and ICP34.5 of Kaposi’s sarcoma-associated herpesvirus (KSHV), human cytomegalovirus (HCMV) and herpes simplex virus (HSV), respectively. (**C**) The early autophagosome recruits the LC-3 protein, a process that is enhanced by VZV, which triggers the maturation of the autophagosome due to the lipidation of LC-3 into LC-3-II. The latter step has been shown to be enhanced by KSHV. (**D**) Ultimately, autophagy concludes with fusion of a mature autophagosome with a lysosome. This process has been shown to be inhibited by VZV and the protein RAB7A by KSHV.

Human herpesviruses have been described to have the capacity to selectively modulate autophagosome formation and generate different cellular outcomes thereof (Figure 2) [5,110,129]. In macrophages, HSV-1 infection causes the formation of autophagosomes, which can process viral proteins prior to their delivery to the proteasome, thus enhancing presentation in MHC-I molecules [130]. On the contrary, HSV-1 ICP34.5 binds to beclin-1, which results in autophagy inhibition in neurons, conferring this virus with neurovirulence properties in mouse models [131]. Furthermore, ICP34.5 has been demonstrated to have an antagonistic function against PKR-induced eIF2α phosphorylation, which aids in preventing autophagy [132] and degrading HSV-1 viral proteins [133]. Through ICP34.5, the inhibition of autophagosome function in dendritic cells allows HSV-1 to prevent viral antigen presentation in MHC-II molecules, thus reducing the capacity of DCs to stimulate and induce the proliferation of CD4+ T cells [134]. ICP34.5 of HSV-2 shares common features with its homolog in HSV-1, such as promoting neurovirulence [89], but for the former, this viral protein allows basal autophagy to occur, which seems to be required for the infection of human corneal epithelial cells [135]. Nevertheless, autophagy is key for enabling protection against HSV-2, as knocking out ATG5 in DCs causes mice to succumb to HSV-2 intravaginal infection [136]. At present, it is currently unknown how viral promotion of the persistence of basal autophagy supports HSV-2 infection and replication.

On the other hand, VZV and EBV potentiate the formation of autophagosomes in virus-infected cells [137]. Indeed, VZV readily induces LC3B (LC3 isoform)-containing autophagosome formation in human skin vesicles, which has been reported to help VZV-related glycoprotein biosynthesis and yield increased viral titers [138]. However, although VZV does not prevent autophagosome formation, it can inhibit late-stage autophagic fluxes, presumably by inhibiting autophagosome and lysosome fusion, which prevents viral degradation and increases viral titers [139]. Nevertheless, it remains yet to be determined what mechanism VZV uses to promote autophagy in infected cells [140,141].

In turn, EBV possesses a latent-lytic switch, which during latent infection activates host PERK and IRE-1 unfolded protein response (UPR) elements while regulating the proliferation of virus-infected B cells [137,142]. Alternatively, the EBV oncoprotein EBNA3C increases autophagy markers in infected cells through the upregulation of tumor suppressor genes, which results in increased survival in transformed B cells [143]. However, increased EBV replication and escape from Burkitt’s lymphoma cells occurs when autophagy is inhibited due to the early lytic gene products BZLF1 and BRLF1 [144]. EBNA1 was observed to accumulate in autophagosomes prior to lysosomal processing for antigen presentation in MHC-II during B cell latent infection [145]. EBV uses an unknown mechanism to downregulate reactive oxygen species (ROS) production in infected cells, with a concomitant reduction in mitochondrial biogenesis, which impairs monocyte differentiation and triggers their apoptosis [146].

Other human herpesviruses appear to mediate mixed-effects over autophagy-related processes. HCMV can primarily induce the autophagosome pathway while inhibiting the degradation of its own newly-synthetized viral proteins by impairing lysosome fusion, promoting the accumulation of LC3 vesicles as a reservoir for viral particle assembly [147]. Indeed, HCMV directs the accumulation of lipidated LC3 and LC3 homologs GABARAPL1- and GATE16-containing vesicles towards the viral assembly complex, along with fragmented Golgi vesicles, in order to aid the cytoplasmic envelopment of viral particles [148]. Moreover, in fibroblasts, HCMV infection inhibited autophagosome formation, either dependently or independently of mTOR signaling [149]. Since mTOR complex 1 is a downstream effector of PI3-kinase signaling, this factor participates in the negative regulation of autophagosome formation. Ultimately, HCMV has been reported to inhibit the completion of autophagy due to the interaction of the TRS1 viral protein with beclin-1, which blocks autophagosome formation [147].

Finally, KSHV has dual effects over autophagy. It has been reported that the viral protein named replication and transcription activator (RTA), which is involved in lytic cycle reactivation from latency, promotes autophagy to facilitate KSHV lytic replication. In addition, KSHV RTA is capable of increasing LC3 lipidated proteins and the number of autophagic vacuoles [150]. Interestingly, during KSHV infection, mTORC1 was required for the synthesis of RTA, but it was unable to affect autophagosome formation or control autophagic flux [151]. Moreover, when the lytic cycle is induced in vitro in latently-infected lymphoma B cells, KSHV, similarly to EBV, promoted autophagy to enhance their replication, while blocking the last autophagic steps by downregulating RAB7A, preventing the fusion of autophagosomes with lysosomes [150,152]. Conversely, KSHV has been reported to have the ability to suppress autophagy by expressing a homolog of FLICE-inhibitor protein vFLIP, which binds to ATG3 and impairs the lipidation and activation of LC3, which is required for autophagosome vacuole formation [153]. Moreover, KSHV encodes a homolog of BCL2, which helps the virus inhibit beclin-1 binding to PI3 kinase complex, thus preventing autophagosome formation [154]. KSHV has also proven to inhibit autophagy in monocytes by de-phosphorylating JNK2, altering the calpains–calpastatin balance and increasing the calpain activity responsible for the cleavage of ATG5 [129]. Furthermore, KSHV also inhibits autophagy in differentiated DCs by hyper-phosphorylating STAT3 [129]. Finally, it is noteworthy to mention that the murine gammaherpesvirus 68 (MHV-68), which is closely related to EBV and KSHV, has been reported to display enhanced reactivation from latency by autophagy-related genes [155].

Overall, autophagy inhibition would help these viruses avoid being recognized by immune components that rely on autophagy-derived class-II antigen presentation [156].

Taken together, although most herpesviruses may lean towards inhibiting autophagosome formation, as it likely represents a threat for virus viability and fitness in the infected cells, other herpesviruses may opt to induce the formation of autophagosomes (Figure 2). Recently, it was reported that HHV-6A promotes autophagy, whereas HHV-6B inhibits autophagy, which was related to their ability to differently modulate the unfolded protein response, impacting cell survival [157]. However, further studies are needed to determine the existing interplay between HHV-6A and HHV-6B viral determinants in autophagosome formation. The effect of HHV-7 infection over autophagy remains to be determined, as well as the potential linkage of autophagy deregulation by this virus and the early onset and development of neurodegenerative diseases [158,159].

## 4. Lysosomal and Golgi-Sorting Vesicles during Herpesvirus Exit

Herpesviruses have been reported to harness vesicular trafficking of the endosomal sorting complex required for transport (ESCRT), responsible for the biogenesis of multivesicular bodies (MVB) [160]. The ESCRT pathway involves the recognition of ubiquitinated cargo and the sorting of membrane vesicles, while vesicles are sculpted to acquire new morphologies and functions such as deforming endosomal membranes to produce MVB [161]. The ESCRT machinery is composed of a set of five protein complexes that are sequentially recruited to late endosomes [162]. Furthermore, it has been described that all ESCRT complexes, namely complexes-0, -I, -II, -III, and the complex formed between Vps4 and Vps-associated protein VTA1, are required to structure the MVB that can then direct cargo to be targeted for lysosomal degradation or exosome formation [163]. Interestingly, MVB plays an important role in transporting glycoproteins to the plasma membrane once they exit the trans-Golgi network [164].

### 4.1. Herpesvirus Modulation of the Lysosomal Pathway

Lysosomal vesicles are a significant portion of the recycling mechanism that processes damaged and senescent macromolecules inside acidic membrane compartments from the cytosol and operates downstream of endosomal trafficking [165]. Importantly, this pathway is also used by the cell for targeting incoming viral particles to degradation as a host antiviral mechanism [166,167].

**Figure 3:**
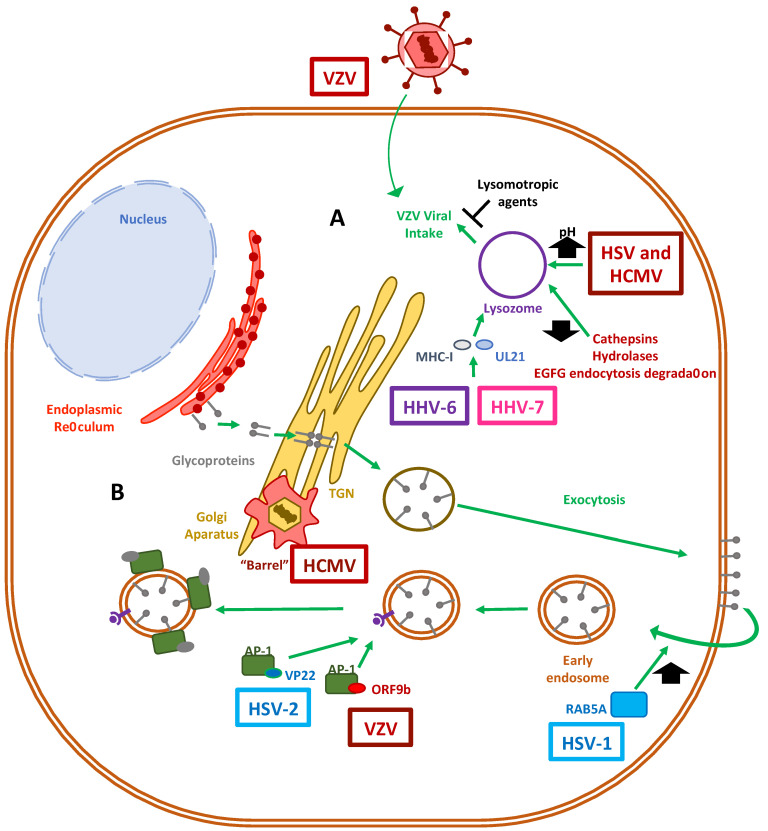
Human herpesvirus infection affects lysosome- and Golgi-sorting vesicle functions. (**A**) Lysosome activity has been shown to be crucial for herpesviruses infection. HSV and HCMV increase the pH levels of lysosomes in order to inactivate them. UL21 from HHV-6 and HHV-7 bind to MHC-I host proteins and divert them toward lysosomal degradation. VZV internalization has been shown to be inhibited by lysomotropic agents, suggesting a key role for the lysosomal pathway in the infection process mediated by this virus. HSV-1 inhibits key steps related to lysosome activation, such as cathepsin maturation, hydrolase activity and the EGFG-mediated endocytic pathway. (**B**) Capsid envelopment and glycoprotein maturation of herpesviruses occur through the Golgi apparatus and the trans-Golgi network (TGN). The corresponding viral proteins are transported to the cell membrane and then internalized into endosomes. This process has been described to be enhanced by means of the RAB5A protein by HSV-1. Clathrin-coated vesicles are transported into the cytosol, where the adapter protein 1 (AP-1) has been shown to interact with the VP22 and ORF9b proteins of HSV-2 and VZV, respectively. HCMV modifies the TGN, forming a barrel-like structure at the cis and trans sides of the Golgi apparatus.

However, HSV-1 and HCMV can survive within lysosomes by increasing the pH of this compartment in order to inactivate lytic enzymes, such as hydrolases, and at the same time by partially acquiring an envelope from these organelles, forming a budding virion (Figure 3) [168]. Moreover, in a neuronal oxidative stress model, HSV-1 was seen to impair some functional features of lysosomes that ranged from an increase in the lysosome load to a reduction in hydrolase activity, as well as the maturation of lysosome cathepsin, along with the inhibition of EGFR-mediated endocytosis degradation [169]. For VZV, it has been reported that intracellular viral uptake by CHO cells is inhibited by lysomotropic agents, which suggests a role for low-pH-dependent endocytic and lysosomal-mediated virus entrance [170].

Additionally, herpesviruses have also been shown to exploit the lysosomal pathway to promote viral maturation (Figure 3). Viral particle maturation may require the budding of virions through intermediary membrane vesicles that compose the interface of the endosomal-lysosomal pathway to travel within the cells [171]. For instance, HSV-1 glycoprotein B has been found in multivesicular bodies (MVB) coming from late endosomes [172]. Of particular interest is the fact that HHV-6A, HHV-6B and HHV-7 all use the viral protein U21 in order to bind to MHC class I molecules and to direct these host proteins needed for antigen presentation to T cells to endolysosomal compartments favoring the evasion of immune system components [173].

Altogether, some herpesviruses can harness the cellular vesicles formed during lysosomal degradation or utilize them to direct glycoproteins to the surface membrane, mediate their transit within the cells and finally mediate the synthesis of new virions. Nevertheless, additional studies are needed in order to better understand the viral determinants that are responsible for modulating lysosomal pathways within cells infected with herpesviruses.

### 4.2. The Role of the Golgi Apparatus in Herpesvirus Virion Maturation

After herpesviruses replicate their genome and assemble their capsids within the nucleus, they are exported from this compartment for further maturation in the cytoplasm. This maturation process involves the addition of several tegument proteins to the capsids, as well as capsid envelopment. The latter requires the transport of viral glycoproteins through vesicles. Herpesvirus glycoproteins converge in the endoplasmic reticulum and then travel to the host cell membrane, before being re-internalized to combine with mature capsids [164]. After the viral glycoproteins reach the Golgi apparatus, they are immersed into the trans-Golgi network (TGN) and are exported to the plasma membrane (Figure 3) [174,175]. Afterward, these proteins return from the cell surface to the cytosol via early endosomes [176] and are combined with viral capsids, thereby creating newly infectious virions within these membranous compartments [177]. This endocytosis step is at least partly mediated by Rab GTPases, such as RAB5A and RAB11, which enable the plasma membranes with viral glycoproteins entering the cell to form endocytic tubular membranes and provide viral capsids with an envelope, as seen in the case of HSV-1 [178]. Regarding anterograde and retrograde endosomal transport of viral particles in neurons, PC12 cells have been described to have viral capsids co-localizing with the trafficking regulator RAB5A, as well as the nerve growth factor NTRK1 (TrkA), which caused a higher expression of the amyloid precursor protein (APP) and altogether could account for the negative implications of some herpesviruses, such as HSV-1 in Alzheimer’s disease [179]. Interestingly, HSV-1 has been shown to induce the fragmentation of the Golgi network upon lytic infection in some cell types, such as Vero and HEp-2 cells [180]. Importantly, the fragments of the Golgi apparatus retained their glycotransferase activities required for O-glycosylation of extracellular viral particles [181].

On the other hand, the tegument protein ORF9b of VZV and its HSV-2 homolog VP22 have been found to interact with adaptor protein 1 (AP-1), which participates in clathrin-coated intracellular vesicle-mediated transport, as well as in defining the cargo of proteins shuttling between endosomes and the trans-Golgi network, and likely contributes to the secondary envelopment stage needed for producing mature herpesvirus virions [182]. Of particular interest was the discovery of the intricate structure of the HCMV assembly complex. This virus reorganizes in an inward barrel architecture at the cis and trans sides of the Golgi network, on top of early endosomal vesicles, and is surrounded by MVBs and recycling endosomes [183]. This process is believed to allow the orchestrated envelopment of herpesviruses before their exit from the cell [184,185]. Finally, the nucleocapsids of HHV-6A and HHV-6B are released from the nucleus into the cytoplasm through a de-envelopment step, where they acquire tegument proteins [186]. This process allows the viruses to egress with a viral membrane ridden with glycoproteins from the trans-Golgi network [187].

Taken together, numerous herpesviruses have evolved strategies for surviving the harsh conditions within lysosomes, and some for benefiting from the ESCRT machinery to travel through MVBs and into the Golgi network [185,188,189]. The modulation of these cellular trafficking pathways underscores the steps required by human herpesviruses to complete effective replication cycles, which result in mature virions that are ready for molecular signals for exiting the infected cell [190].

## 5. Exocytosis Vesicles Hijacked by Herpesviruses

**Figure 4:**
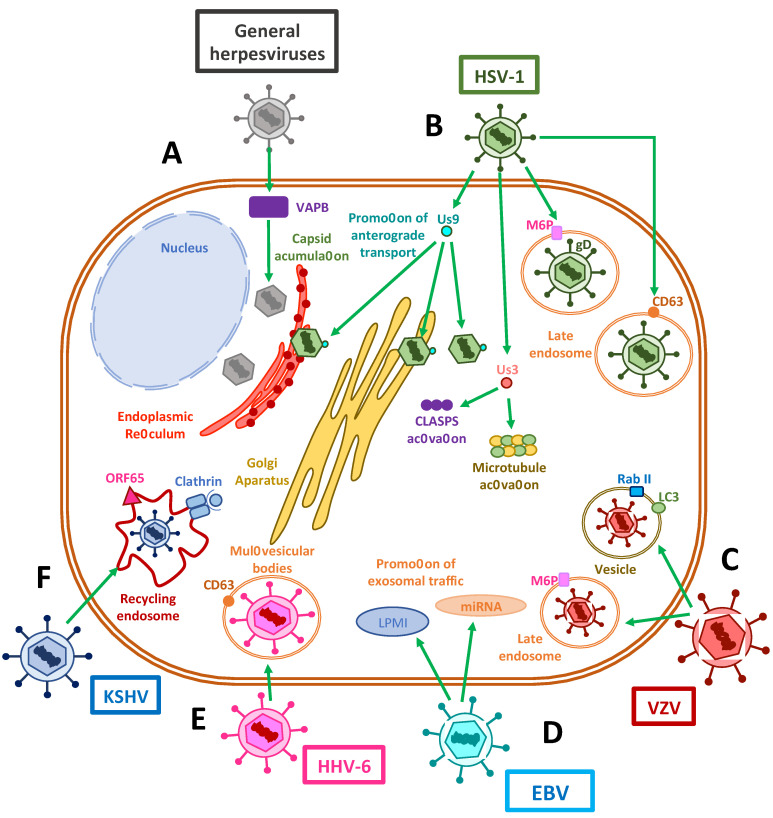
Exocytosis vesicles participate in herpesvirus exit. (**A**) The accumulation of herpesvirus capsids in the cytoplasm has been shown to be assisted by VAPB host proteins. (**B**) The HSV-1 Us9 protein interacts with capsids in the endoplasmic reticulum (ER), the Golgi apparatus and the cytosol in order to promote the anterograde transport of viral components through microtubules. Us3 protein has also been shown to stabilize and activate microtubules and CLASP complexes to promote vesicle transport. The interactions of viral gD with host M6P and its receptors promote the export of virus-containing late endosomes. On the other hand, viral proteins have been shown to co-localize with CD63 or CD63 and MHC-II, suggesting a potential immune-modulatory role for late endosomal trafficking. (**C**) VZV has been reported to use M6P and its receptors to promote viral export. Additionally, this virus appears to be exocytosed in single-membrane vesicles from the autophagosomal pathway, which contain LC-3 and Rab II. (**D**) EBV shuttles LPMI- and miRNA-containing vesicles derived in exosomal traffic modulation. (**E**) Exit of HHV-6 occurs through the exosomal pathway, using multivesicular bodies (MVBs). (**F**) Finally, KSHV recycles clathrin-coated endosomal vesicles, used during cell entry, for viral exit using the viral protein ORF65.

To allow the egress of newly-synthesized infectious herpesvirus particles from the infected cells, the viral capsids that are assembled in the nucleus must travel through the perinuclear space, through the inner nuclear membrane (INM) and outer nuclear membrane (ONM) to reach the cytoplasm, followed by their envelopment within vesicles containing the viral glycoproteins that decorate the viral envelope [175,191]. After an initial envelopment at the INM, the enveloped capsids are released into the perinuclear space, from which the virus fuses to the ONM and releases into the cytosol the viral capsids [192,193]. These capsids are then reenveloped in vesicles that belong to the trans-Golgi network, or in the case of HCMV, enter secretory pathway-derived vesicles from the virus assembly complex (vAC) for their extracellular release as enveloped virions within exocytosis vesicles (Figure 4) [184,194].

### 5.1. Modulation of Exocytosis by Herpesvirus

Interestingly, several viral glycoproteins that are not found in fully developed and mature extracellular virions are involved in capsid egress from the perinuclear space. Among these proteins are pUL31, which is a nuclear matrix-associated phosphoprotein; pUL34, a nuclear membrane-associated phosphoprotein; and Us3, a serine/threonine kinase that interacts with lamins A and C to disrupt the nuclear lamina in order to promote the envelopment of nascent viral capsids [192,195,196]. More recently, it has been reported that depletion of the host vesicle-associated membrane protein-associated protein B (VAPB) caused the accumulation of viral capsids in the nucleus, which supports its involvement in primary envelopment during nuclear egress [197]. Furthermore, the HSV-1 envelope protein US9 has been shown to associate with capsids in the cytoplasm, the endoplasmic reticulum and the Golgi apparatus, and to function in concert with gE/gI during anterograde transport within axons in neurons by promoting both the loading of capsids and glycoprotein-containing vesicles onto kinesin motors and microtubules [198].

Notably, several viral molecules participate in the active transport of newly synthesized virions through the exocytic route. Similar to the interaction of tegument proteins in the transport of capsids during cell entry, UL36 and UL37 proteins may be involved in plus-end capsid transport towards the cell surface [199]. For example, UL20, an envelope protein that is highly conserved in alphaherpesviruses, has been implicated in syncytia formation, which is required for cell-to-cell infection, and also for cytoplasmic envelopment within the trans-Golgi network (TGN) and for virion transport from the TGN to the plasma membrane or extracellular space, therefore promoting the egress of infectious virions [200]. Importantly, it has been found that the function of UL20 of HSV depends on its localization at the Golgi apparatus, causing it to be palmitoylated by host GODZ, which allows the proper trafficking of UL20 and gK to exocytosis vesicles [201]. Here, they would participate, along with gM, in incorporating other glycoproteins such as gD and gH/gL into mature virions [202].

Additionally, herpesviruses can stabilize microtubules to enhance virus release [203]. For instance, the HSV-1 protein US3 has been reported to stabilize microtubule structures and be necessary for viral spread by activating cytoplasmic linker-associated proteins (CLASPs), a type of plus-end tracking protein (+TIP) that operates in microtubule nucleation at the Golgi apparatus and captures microtubules near the membrane bilayer [204]. HSV-1 employs a mechanism in which glycoproteins such as gD are modified with mannose-6-phosphate [205] residues that cause their export within late endosomes containing M6P receptors through the TGN, and which culminates in viral assembly complexes for virus egress [205].

### 5.2. Modulation of Endosomal and Exosomal Pathways by Herpesviruses

Herpesviruses have also evolved molecular mechanisms to interfere with the endosomal-exosomal pathways of infected cells (Figure 4). For instance, gB of HSV-1 is frequently found in extracellular vesicles and has been shown to co-localize with the late host endosomal marker CD63 and bind to MHC-II molecules (HLA-DR in human cell lines) in such a way to delay its endosomal trafficking, which may have immunomodulatory effects [206,207]. Furthermore, exosomal targeting, but not MHC-II interference, is mostly conserved in other gB-bearing alphaherpesviruses [208]. This finding may prompt investigations into how the exosomal release of gB improves viral fitness while roaming in the infected host. On the other hand, VZV utilizes an amalgam of endosomal and autophagosomal pathways to perform exocytosis of complete viral particles, as observed by electron microscopy, which shows virions in single-membraned vesicles carrying both Rab11 and LC3, which resembled amphisomes [209]. Alternatively, VZV has been shown to have the ability to bind to the mannose-6-phosphate receptor in late endosomes as a mechanism to egress from infected melanoma cells or fibroblasts, regardless of the autophagosome pathway [210]. A recent report indicates that the VZV glycoprotein M (gM) contains two tyrosine-XX-bulk hydrophobic amino acid domains and one dileucine trafficking-domain associated with trans-Golgi sorting vesicles, which implies that gM is targeted to endosomal-lysosomal compartments to help viral maturation [211]. Interestingly, manipulating these motifs in VZV mutants did not impair virus assembly or egress, but rather increased its virulence in the skin in a humanized severe combined immune deficient (SCID) mouse model [211]. The actual mechanism by which the glycoprotein M is responsible for increased virulence is currently unknown.

EBV employs the exosomal pathway to introduce several microRNAs and LMP-1 in extracellular vesicles, capable of altering the latent-lytic cycle of an infected cell [212,213]. The latter feature likely promotes the communication between epithelial cells undergoing lytic replication to direct the maintenance of a latently-infected B cell [213]. To this end, CD63 has been found to promote LMP-1 exosomal trafficking and enhance exosome production [214].

Meanwhile, as discussed above, HCMV has an organized assembly complex in which after capsids acquire their tegument layer, they pass through the TGN to acquire an envelope and likely obtain the necessary glycoproteins from early endosomes, which are ultimately released from recycling endosomes or Golgi-derived membranes [215,216].

Regarding HHV-6, previous reports indicate that viral glycoproteins co-localize with CD63, a marker of late endosomes that is found in MVB vesicles, suggesting that the infectious viruses are released by the cell via the exosomal pathway. Noteworthily, it is currently unknown what processes are involved during HHV-7 virion exit [187].

In addition to playing an active role in KSHV during entry, actin filaments also undergo rearrangements that drive the trafficking of viral proteins throughout the cytosol of endothelial cells, as seen with ORF65, through clathrin-coated early and recycled endosomes during secondary envelopment steps [107].

Altogether, at present there is relatively scarce information available regarding the multiple processes by which herpesvirus may prefer to leave infected cells and how viral-derived exosomes affect neighboring cells. For example, it is unknown whether most herpesviruses possess a mechanism for egressing similarly to HSV-1, using a UL20/gK/gM complex directing viral glycoproteins to exocytosis vesicles to assemble mature virions [203]. Therefore, additional studies are needed to elucidate how herpesviruses egress infected cells and to explore the pros and cons of these routes.

## 6. Concluding Remarks

Herpesviruses use and modulate several host intracellular membrane trafficking pathways in order to elicit productive infection, effective virus replication and synthesis of new infectious viral particles and, finally, virion exit from the infected cells. Although significant progress has been made regarding the understanding of the interplay between herpesviruses and their host, namely at the cellular level, increased knowledge is required in this respect, which could shed light on novel strategies for their control or inhibition. Particularly, much remains to be studied in relation to viruses such as HHV-6 and HHV-7, regarding what viral components mediate and modulate host membrane vesicle trafficking, their interactions and the key factors in the infectious cycles of these viruses. In particular, future work may focus on how intracellular viral particles are differentially directed to specific cell membranes or sites within the cell for exocytosis or cell-to-cell spread.

Additional studies are needed in order to determine the roles of each of the viral and host factors that mediate the processes involving intracellular host membranes that are modulated during the replication cycle of herpesviruses. Again, knowledge in this regard should contribute to the understanding of critical interactions that could be potentially targeted by different drugs to limit viral replication. Furthermore, we foresee that new studies providing insights into the workings and regulations of the vast intracellular transport machinery and how it assists viral infections will be valuable in the long-term for harnessing herpesviruses and limiting the diseases they produce.

## Data Availability

No new data were created or analyzed in this study. Data sharing is not applicable to this article.

## References

[1] Rajsbaum R., García-Sastre A. (2013). Viral evasion mechanisms of early antiviral responses involving regulation of ubiquitin pathways. Trends Microbiol..

[2] Yamauchi Y., Helenius A. (2013). Virus entry at a glance. J. Cell Sci..

[3] Lee H.-C., Chathuranga K., Lee J.-S. (2019). Intracellular sensing of viral genomes and viral evasion. Exp. Mol. Med..

[4] Tognarelli E.I., Palomino T.F., Corrales N., Bueno S.M., Kalergis A.M., González P.A. (2019). Herpes Simplex Virus Evasion of Early Host Antiviral Responses. Front. Cell. Infect. Microbiol..

[5] Taylor M.P., Koyuncu O.O., Enquist L.W. (2011). Subversion of the actin cytoskeleton during viral infection. Nat. Rev. Genet..

[6] Bär S., Rommelaere J., Nüesch J.P.F. (2013). Vesicular Transport of Progeny Parvovirus Particles through ER and Golgi Regulates Maturation and Cytolysis. PLoS Pathog..

[7] Votteler J., Sundquist W.I. (2013). Virus Budding and the ESCRT Pathway. Cell Host Microbe.

[8] Pocock G.M., Becker J.T., Swanson C.M., Ahlquist P., Sherer N.M. (2016). HIV-1 and M-PMV RNA Nuclear Export Elements Program Viral Genomes for Distinct Cytoplasmic Trafficking Behaviors. PLoS Pathog..

[9] Ravindran M.S., Bagchi P., Cunningham C.N., Tsai B. (2016). Opportunistic intruders: How viruses orchestrate ER functions to infect cells. Nat. Rev. Genet..

[10] Münz C. (2017). The Autophagic Machinery in Viral Exocytosis. Front. Microbiol..

[11] Robinson M., Schor S., Barouch-Bentov R., Einav S. (2018). Viral journeys on the intracellular highways. Cell. Mol. Life Sci..

[12] Knoops K., Kikkert M., Van Den Worm S.H., Zevenhoven-Dobbe J.C., Van Der Meer Y., Koster A.J. (2008). SARS-coronavirus replication is supported by a reticulovesicular network of modified endoplasmic reticulum. PLoS Biol..

[13] Amorim M.J., Bruce E.A., Read E.K., Foeglein Á., Mahen R., Stuart A.D., Digard P. (2011). A Rab11- and microtu-bule-dependent mechanism for cytoplasmic transport of influenza A virus viral RNA. J. Virol..

[14] Schiffer J.T., Swan D.A., Corey L., Wald A. (2013). Rapid viral expansion and short drug half-life explain the incom-plete effectiveness of current herpes simplex virus 2-directed antiviral agents. Antimicrob. Agents Chemother..

[15] Ju X., Yan Y., Liu Q., Li N., Sheng M., Zhang L. (2015). Neuraminidase of Influenza A Virus Binds Lysosome-Associated Membrane Proteins Directly and Induces Lysosome Rupture. J. Virol..

[16] Barouch-Bentov R., Neveu G., Xiao F., Beer M., Bekerman E., Schor S., Campbell J., Boonyaratanakornkit J., Lindenbach B., Lu A. (2016). Hepatitis C Virus Proteins Interact with the Endosomal Sorting Complex Required for Transport (ESCRT) Machinery via Ubiquitination To Facilitate Viral Envelopment. mBio.

[17] Oliver S.L., Zhou M., Arvin A.M. (2020). Varicella-zoster virus: Molecular controls of cell fusion-dependent pathogenesis. Biochem. Soc. Trans..

[18] Nowalk A., Green M. (2016). Epstein-Barr Virus. Microbiol. Spectr..

[19] Iizasa H., Nanbo A., Nishikawa J., Jinushi M., Yoshiyama H. (2012). Epstein-Barr Virus (EBV)-associated gastric carcinoma. Viruses.

[20] Griffiths P., Baraniak I., Reeves M. (2015). The pathogenesis of human cytomegalovirus. J. Pathol..

[21] Dollard S.C., Grosse S.D., Ross D.S. (2007). New estimates of the prevalence of neurological and sensory sequelae and mortality associated with congenital cytomegalovirus infection. Rev. Med. Virol..

[22] Marsico C., Kimberlin D.W. (2017). Congenital Cytomegalovirus infection: Advances and challenges in diagnosis, prevention and treatment. Ital. J. Pediatr..

[23] Agut H., Bonnafous P., Gautheret-Dejean A. (2015). Laboratory and Clinical Aspects of Human Herpesvirus 6 Infections. Clin. Microbiol. Rev..

[24] Mori Y., Yamanishi K. (2007). HHV-6A, 6B, and 7: Pathogenesis, Host Response, and Clinical Disease. Human Herpesviruses: Biology, Therapy, and Immunoprophylaxis.

[25] Wolz M.M., Sciallis G.F., Pittelkow M.R. (2012). Human Herpesviruses 6, 7, and 8 from a Dermatologic Perspective. Mayo Clin. Proc..

[26] Mesri E.A., Cesarman E., Boshoff C. (2010). Kaposi’s sarcoma and its associated herpesvirus. Nat. Rev. Cancer.

[27] Sir D., Ou J.-H.J. (2010). Autophagy in viral replication and pathogenesis. Mol. Cells.

[28] Ahmad I., Wilson D.W. (2020). HSV-1 Cytoplasmic Envelopment and Egress. Int. J. Mol. Sci..

[29] Conner S.D., Schmid S.L. (2003). Regulated portals of entry into the cell. Nat. Cell Biol..

[30] Kaksonen M., Roux A. (2018). Mechanisms of clathrin-mediated endocytosis. Nat. Rev. Mol. Cell Biol..

[31] Aderem A., Underhill D.M. (1999). Mechanisms of phagocytosis in macrophages. Annu. Rev. Immunol..

[32] Adok V.A., Chimini G. (2001). The phagocytosis of apoptotic cells. Semin. Immunol..

[33] Mayor S., Parton R.G., Donaldson J.G. (2014). Clathrin-Independent Pathways of Endocytosis. Cold Spring Harb. Perspect. Biol..

[34] Conner S.D., Schmid S.L. (2003). Differential requirements for AP-2 in clathrin-mediated endocytosis. J. Cell Biol..

[35] Sandvig K., Pust S., Skotland T., Van Deurs B. (2011). Clathrin-independent endocytosis: Mechanisms and function. Curr. Opin. Cell Biol..

[36] Canton J. (2018). Macropinocytosis: New Insights into Its Underappreciated Role in Innate Immune Cell Surveillance. Front. Immunol..

[37] Parton R.G., Del Pozo M.A., Vassilopoulos S., Nabi I.R., Le Lay S., Lundmark R., Kenworthy A.K., Camus A., Blouin C.M., Sessa W.C. (2020). Caveolae: The FAQs. Traffic.

[38] Sodeik B., Ebersold M.W., Helenius A. (1997). Microtubule-mediated Transport of Incoming Herpes Simplex Virus 1 Capsids to the Nucleus. J. Cell Biol..

[39] Suazo P.A., Ibañez F.J., Retamal-Díaz A.R., Paz-Fiblas M.V., Bueno S.M., Kalergis A.M., González P.A. (2015). Evasion of early antiviral responses by herpes simplex viruses. Mediat. Inflamm..

[40] Nicola A.V., McEvoy A.M., Straus S.E. (2003). Roles for Endocytosis and Low pH in Herpes Simplex Virus Entry into HeLa and Chinese Hamster Ovary Cells. J. Virol..

[41] Nicola A.V., Straus S.E., Bubić I., Wagner M., Krmpotić A., Saulig T., Kim S., Yokoyama W.M., Jonjić S., Koszinowski U.H. (2004). Cellular and Viral Requirements for Rapid Endocytic Entry of Herpes Simplex Virus. J. Virol..

[42] Akhtar J., Shukla D. (2009). Viral entry mechanisms: Cellular and viral mediators of herpes simplex virus entry. FEBS J..

[43] Mercer J., Schelhaas M., Helenius A. (2010). Virus Entry by Endocytosis. Annu. Rev. Biochem..

[44] Brodsky F.M. (2012). Diversity of Clathrin Function: New Tricks for an Old Protein. Annu. Rev. Cell Dev. Biol..

[45] Brodsky F.M., Chen C.-Y., Knuehl C., Towler M.C., Wakeham D.E. (2001). Biological Basket Weaving: Formation and Function of Clathrin-Coated Vesicles. Annu. Rev. Cell Dev. Biol..

[46] Kirchhausen T., Owen D., Harrison S.C. (2014). Molecular structure, function, and dynamics of clathrin-mediated membrane traffic. Cold Spring Harb. Perspect. Biol..

[47] Akamatsu M., Vasan R., Serwas D., Ferrin M.A., Rangamani P., Drubin D.G. (2020). Principles of self-organization and load adaptation by the actin cytoskeleton during clathrin-mediated endocytosis. Elife.

[48] Zhang B., Zelhof A.C. (2002). Amphiphysins: Raising the BAR for Synaptic Vesicle Recycling and Membrane Dynamics. Traffic.

[49] Simunovic M., Manneville J.-B., Renard H.-F., Evergren E., Raghunathan K., Bhatia D., Kenworthy A.K., Voth G.A., Prost J., McMahon H.T. (2017). Friction Mediates Scission of Tubular Membranes Scaffolded by BAR Proteins. Cell.

[50] Herold B.C., Visalli R.J., Susmarski N., Brandt C.R., Spear P.G. (1994). Glycoprotein C-independent binding of herpes simplex virus to cells requires cell surface heparan sulphate and glycoprotein B. J. Gen. Virol..

[51] Gerber S.I., Belval B.J., Herold B.C. (1995). Differences in the role of glycoprotein C of HSV-1 and HSV-2 in viral binding may contribute to serotype differences in cell tropism. Virology.

[52] Satoh T., Arii J., Suenaga T., Wang J., Kogure A. (2008). PILRalpha is a herpes simplex virus-1 entry coreceptor that associates with glycoprotein B. Cell.

[53] Martinez W.M., Spear P.G. (2001). Structural Features of Nectin-2 (HveB) Required for Herpes Simplex Virus Entry. J. Virol..

[54] Stiles K.M., Whitbeck J.C., Lou H., Cohen G.H., Eisenberg R.J., Krummenacher C. (2010). Herpes simplex virus gly-coprotein D interferes with binding of herpesvirus entry mediator to its ligands through downregulation and direct com-petition. J. Virol..

[55] Karaba A.H., Kopp S.J., Longnecker R. (2011). Herpesvirus entry mediator and nectin-1 mediate herpes simplex virus 1 infection of the murine cornea. J. Virol..

[56] Montgomery R., Warner M.S., Lum B.J., Spear P.G. (1996). Herpes Simplex Virus-1 Entry into Cells Mediated by a Novel Member of the TNF/NGF Receptor Family. Cell.

[57] Backovic M., Jardetzky T.S. (2011). Class III Viral Membrane Fusion Proteins. Adv. Exp. Med. Biol..

[58] Cairns T.M., Whitbeck J.C., Lou H., Heldwein E.E., Chowdary T.K., Eisenberg R.J., Cohen G.H. (2011). Capturing the herpes simplex virus core fusion complex (gB-gH/gL) in an acidic environment. J. Virol..

[59] Silverman J.L., Heldwein E.E. (2013). Mutations in the cytoplasmic tail of herpes simplex virus 1 gH reduce the fuso-genicity of gB in transfected cells. J. Virol..

[60] Dollery S.J., Wright C.C., Johnson D.C., Nicola A.V. (2011). Low-pH-dependent changes in the conformation and oligomeric state of the prefusion form of herpes simplex virus glycoprotein B are separable from fusion activity. J. Virol..

[61] Muggeridge M.I. (2012). Glycoprotein B of Herpes Simplex Virus 2 Has More than One Intracellular Conformation and Is Altered by Low pH. J. Virol..

[62] Sari T.K., Gianopulos K.A., Weed D.J., Schneider S.M., Pritchard S.M., Nicola A.V. (2020). Herpes Simplex Virus Glycoprotein C Regulates Low-pH Entry. mSphere.

[63] Crisci E., Ellegård R., Nyström S., Rondahl E., Serrander L., Bergström T., Sjöwall C., Eriksson K., Larsson M. (2016). Complement Opsonization Promotes Herpes Simplex Virus 2 Infection of Human Dendritic Cells. J. Virol..

[64] Zerboni L., Sen N., Oliver S.L., Arvin A.M. (2014). Molecular mechanisms of varicella zoster virus pathogenesis. Nat. Rev. Genet..

[65] Suenaga T., Satoh T., Somboonthum P., Kawaguchi Y., Mori Y., Arase H. (2009). Myelin-associated glycoprotein mediates membrane fusion and entry of neurotropic herpesviruses. Proc. Natl. Acad. Sci. USA.

[66] Chen J.J., Zhu Z., Gershon A.A., Gershon M.D. (2004). Mannose 6-Phosphate Receptor Dependence of Varicella Zoster Virus Infection In Vitro and in the Epidermis during Varicella and Zoster. Cell.

[67] Pasieka T.J., Maresova L., Grose C. (2003). A Functional YNKI Motif in the Short Cytoplasmic Tail of Varicella-Zoster Virus Glycoprotein gH Mediates Clathrin-Dependent and Antibody-Independent Endocytosis. J. Virol..

[68] Pasieka T.J., Maresova L., Shiraki K., Grose C. (2004). Regulation of Varicella-Zoster Virus-Induced Cell-to-Cell Fusion by the Endocytosis-Competent Glycoproteins gH and gE. J. Virol..

[69] Shannon-Lowe C., Adland A.I., Bell H.J., Delecluse A.B. (2009). Features distinguishing Ep-stein-Barr virus infections of epithelial cells and B cells: Viral genome expression, genome maintenance, and genome am-plification. J. Virol..

[70] Chen J., Sathiyamoorthy K., Zhang X., Schaller S., White B.E.P., Jardetzky T.S., Longnecker R. (2018). Ephrin receptor A2 is a functional entry receptor for Epstein–Barr virus. Nat. Microbiol..

[71] Tugizov S.M., Berline J.W., Palefsky J.M. (2003). Epstein-Barr virus infection of polarized tongue and nasopharyngeal epithelial cells. Nat. Med..

[72] Chesnokova L.S., Nishimura S.L., Hutt-Fletcher L.M. (2009). Fusion of epithelial cells by Epstein-Barr virus proteins is triggered by binding of viral glycoproteins gHgL to integrins alphavbeta6 or alphavbeta8. Proc. Natl. Acad. Sci. USA.

[73] Wang H.-B., Zhang H., Zhang J.-P., Li Y., Zhao B., Feng G.-K., Du Y., Xiong D., Zhong Q., Liu W.-L. (2015). Neuropilin 1 is an entry factor that promotes EBV infection of nasopharyngeal epithelial cells. Nat. Commun..

[74] Miller N., Hutt-Fletcher L.M. (1992). Epstein-Barr virus enters B cells and epithelial cells by different routes. J. Virol..

[75] Speck P., Haan K.M., Longnecker R. (2000). Epstein-Barr virus entry into cells. Virology.

[76] Kirschner A.N., Omerović J., Popov B., Longnecker R., Jardetzky T.S. (2006). Soluble Epstein-Barr virus glycoproteins gH, gL, and gp42 form a 1:1:1 stable complex that acts like soluble gp42 in B-cell fusion but not in epithelial cell fusion. J. Virol..

[77] Kirschner A.N., Sorem J., Longnecker R., Jardetzky T.S. (2009). Structure of Epstein-Barr virus glycoprotein 42 suggests a mechanism for triggering receptor-activated virus entry. Structure.

[78] Compton T., Nowlin D.M., Cooper N.R. (1993). Initiation of human cytomegalovirus infection requires initial inter-action with cell surface heparan sulfate. Virology.

[79] Carlson C., Britt W.J., Compton T. (1997). Expression, Purification, and Characterization of a Soluble Form of Human Cytomegalovirus Glycoprotein B. Virology.

[80] Viswanathan K., Smith M.S., Malouli D., Mansouri M., Nelson J.A., Früh K. (2011). BST2/Tetherin Enhances Entry of Human Cytomegalovirus. PLoS Pathog..

[81] Soroceanu L., Akhavan A., Cobbs C.S. (2008). Platelet-derived growth factor-α receptor activation is required for human cytomegalovirus infection. Nat. Cell Biol..

[82] Feire A.L., Koss H., Compton T. (2004). Cellular integrins function as entry receptors for human cytomegalovirus via a highly conserved disintegrin-like domain. Proc. Natl. Acad. Sci. USA.

[83] Wang X.D.Y., Huang S.M. (2005). Integrin alphavbeta3 is a coreceptor for human cytomegalovi-rus. Nat. Med..

[84] Wang D., Shenk T. (2005). Human cytomegalovirus virion protein complex required for epithelial and endothelial cell tropism. Proc. Natl. Acad. Sci. USA.

[85] Ryckman B.J., Rainish B.L., Chase M.C., Borton J.A., Nelson J.A., Jarvis M.A., Johnson D.C. (2007). Characterization of the Human Cytomegalovirus gH/gL/UL128-131 Complex That Mediates Entry into Epithelial and Endothelial Cells. J. Virol..

[86] Ryckman B.J., Jarvis M.A., Drummond D.D., Nelson J.A., Johnson D.C. (2006). Johnson Human cytomegalovirus entry into epithelial and endothelial cells depends on genes UL128 to UL150 and occurs by endocytosis and low-pH fusion. J. Virol..

[87] Meraner P., Lu P., Perreira J.M., Aker A.M., McDougall W.M., Zhuge R., Chan G.C., Gerstein R.M., Caposio P. (2019). OR14I1 is a receptor for the human cytomegalovirus pentameric complex and defines viral epithelial cell tropism. Proc. Natl. Acad. Sci. USA.

[88] Tang H., Hayashi M., Maeki T., Yamanishi K., Mori Y. (2011). Human Herpesvirus 6 Glycoprotein Complex Formation Is Required for Folding and Trafficking of the gH/gL/gQ1/gQ2 Complex and Its Cellular Receptor Binding. J. Virol..

[89] Tang H., Serada S., Kawabata A., Ota M., Hayashi E., Naka T., Yamanishi K., Mori Y. (2013). CD134 is a cellular receptor specific for human herpesvirus-6B entry. Proc. Natl. Acad. Sci. USA.

[90] Mori Y., Yang X., Akkapaiboon P., Okuno T., Yamanishi K. (2003). Human Herpesvirus 6 Variant A Glycoprotein H-Glycoprotein L-Glycoprotein Q Complex Associates with Human CD46. J. Virol..

[91] Akkapaiboon P., Mori Y., Sadaoka T., Yonemoto S., Yamanishi K. (2004). Intracellular Processing of Human Herpesvirus 6 Glycoproteins Q1 and Q2 into Tetrameric Complexes Expressed on the Viral Envelope. J. Virol..

[92] Maeki T., Hayashi M., Kawabata A., Tang H., Yamanishi K., Mori Y. (2013). Identification of the Human Herpesvirus 6A gQ1 Domain Essential for Its Functional Conformation. J. Virol..

[93] Tanaka Y., Suenaga T., Matsumoto M., Seya T., Arase H. (2013). Herpesvirus 6 Glycoproteins B (gB), gH, gL, and gQ Are Necessary and Sufficient for Cell-to-Cell Fusion. J. Virol..

[94] Nii S., Yoshida M., Uno F., Kurata T., Ikuta K., Yamanishi K. (1990). Replication of Human Herpesvirus 6 (HHV-6): Morphological Aspects. Adv. Exp. Med. Biol..

[95] Cirone M., Zompetta C., Angeloni A., Ablashi D., Salahuddin S., Pavan A., Torrisi M., Frati L., Faggioni A. (1992). Infection by Human Herpesvirus 6 (HHV-6) of Human Lymphoid T Cells Occurs Through an Endocytic Pathway. AIDS Res. Hum. Retrovir..

[96] Tang H., Kawabata A., Takemoto M., Yamanishi K., Mori Y. (2008). Human herpesvirus-6 infection induces the reor-ganization of membrane microdomains in target cells, which are required for virus entry. Virology.

[97] Ahlqvist J., Donati D., Martinelli E., Akhyani N., Hou J., Major E.O., Jacobson S., Fogdell-Hahn A. (2006). Complete replication cycle and acquisition of tegument in nucleus of human herpesvirus 6A in astrocytes and in T-cells. J. Med. Virol..

[98] Secchiero P., Sun D., De Vico A.L., Crowley R.W., Reitz M.S., Zauli G., Lusso P., Gallo R.C. (1997). Role of the extracellular domain of human herpesvirus 7 glycoprotein B in virus binding to cell surface heparan sulfate proteoglycans. J. Virol..

[99] Skrincosky D., Hocknell P., Whetter L., Secchiero P., Chandran B., Dewhurst S. (2000). Identification and Analysis of a Novel Heparin-Binding Glycoprotein Encoded by Human Herpesvirus 7. J. Virol..

[100] Lusso P., Secchiero P., Crowley R.W., Garzino-Demo A., Berneman Z.N., Gallo R.C. (1994). CD4 is a critical compo-nent of the receptor for human herpesvirus 7: Interference with human immunodeficiency virus. Proc. Natl. Acad. Sci. USA.

[101] Su C., Wu L., Chai Y., Qi J., Tan S., Gao G.F., Song H., Yan J. (2020). Molecular basis of EphA2 recognition by gHgL from gammaherpesviruses. Nat. Commun..

[102] Hahn A.S., Desrosiers R.C. (2014). Binding of the Kaposi’s sarcoma-associated herpesvirus to the ephrin binding surface of the EphA2 receptor and its inhibition by a small molecule. J. Virol..

[103] Chen J., Zhang X., Schaller S., Jardetzky T.S., Longnecker R. (2019). Ephrin Receptor A4 is a New Kaposi’s Sar-coma-Associated Herpesvirus Virus Entry Receptor. mBio.

[104] Raghu H., Sharma-Walia N., Veettil M.V., Sadagopan S., Chandran B. (2009). Kaposi’s sarcoma-associated herpesvirus utilizes an actin polymerization-dependent macropinocytic pathway to enter human dermal microvascular endothelial and human umbilical vein endothelial cells. J. Virol..

[105] Chakraborty S.M., ValiyaVeettil S., Sadagopan N., Chandran B. (2011). c-Cbl-mediated selective virus-receptor translocations into lipid rafts regulate productive Kaposi’s sarcoma-associated herpesvirus infection in endothelial cells. J. Virol..

[106] Veettil M.V., Sadagopan S., Kerur N., Chakraborty S., Chandran B. (2010). Chakraborty and B. Chandran Interaction of c-Cbl with myosin IIA regulates Bleb associated macropinocytosis of Kaposi’s sarcoma-associated herpesvirus. PLoS Pathog..

[107] Greene W., Gao S.J. (2009). Actin dynamics regulate multiple endosomal steps during Kaposi’s sarcoma-associated herpesvirus entry and trafficking in endothelial cells. PLoS Pathog..

[108] Zheng K., Xiang Y., Wang X., Wang Q., Zhong M., Wang S. (2014). Epidermal growth fac-tor receptor-PI3K signaling controls cofilin activity to facilitate herpes simplex virus 1 entry into neuronal cells. mBio.

[109] Shoji-Kawata S., Levine B. (2009). Autophagy, antiviral immunity, and viral countermeasures. Biochim. Biophys. Acta BBA Bioenerg..

[110] Ahmad L., Mostowy S., Sancho-Shimizu V. (2018). Autophagy-Virus Interplay: From Cell Biology to Human Disease. Front. Cell Dev. Biol..

[111] . Valečka J., Almeida C.R., Su B., Pierre P., Gatti E. (2018). Autophagy and MHC-restricted antigen presentation. Mol. Immunol..

[112] Van Kaer L., Parekh V.V., Postoak J.L., Wu L. (2019). Role of autophagy in MHC class I-restricted antigen presentation. Mol. Immunol..

[113] Schmid D., Pypaert M., Münz C. (2007). Antigen-Loading Compartments for Major Histocompatibility Complex Class II Molecules Continuously Receive Input from Autophagosomes. Immunity.

[114] Carlsson S.R., Simonsen A. (2015). Membrane dynamics in autophagosome biogenesis. J. Cell Sci..

[115] Mizushima N., Yoshimori T., Ohsumi Y. (2011). The Role of Atg Proteins in Autophagosome Formation. Annu. Rev. Cell Dev. Biol..

[116] Xie Z., Klionsky D.J. (2007). Autophagosome formation: Core machinery and adaptations. Nat. Cell Biol..

[117] Furuya N., Yu J., Byfield M., Pattingre S., Levine B. (2005). The evolutionarily conserved domain of Beclin 1 is required for Vps34 binding, autophagy and tumor suppressor function. Autophagy.

[118] Menon M.B., Dhamija S. (2018). Beclin 1 Phosphorylation—At the Center of Autophagy Regulation. Front. Cell Dev. Biol..

[119] Axe E.L., Walker S.A., Manifava M., Chandra P., Roderick H.L., Habermann A., Griffiths G., Ktistakis N.T. (2008). Autophagosome formation from membrane compartments enriched in phosphatidylinositol 3-phosphate and dynamically connected to the endoplasmic reticulum. J. Cell Biol..

[120] Otomo C., Metlagel Z., Takaesu G., Otomo T. (2013). Structure of the human ATG12~ATG5 conjugate required for LC3 lipidation in autophagy. Nat. Struct. Mol. Biol..

[121] Kabeya Y., Mizushima N., Ueno T., Yamamoto A., Kirisako T., Noda T. (2000). LC3, a mammalian homologue of yeast Apg8p, is localized in autophagosome membranes after processing. EMBO J..

[122] Agrotis A., Pengo N., Burden J.J., Ketteler R. (2019). Redundancy of human ATG4 protease isoforms in autophagy and LC3/GABARAP processing revealed in cells. Autophagy.

[123] Ichimura Y., Kirisako T., Takao T., Satomi Y., Shimonishi Y., Ishihara N., Ohsumi Y. (2000). A ubiquitin-like system mediates protein lipidation. Nature.

[124] Yang Z., Klionsky D.J. (2010). Mammalian autophagy: Core molecular machinery and signaling regulation. Curr. Opin. Cell Biol..

[125] Frudd K., Burgoyne T., Burgoyne J.R. (2018). Oxidation of Atg3 and Atg7 mediates inhibition of autophagy. Nat. Commun..

[126] Nair U., Jotwani A., Geng J., Gammoh N., Richerson D., Yen W.-L., Griffith J., Nag S., Wang K., Moss T. (2011). SNARE Proteins Are Required for Macroautophagy. Cell.

[127] Diao J., Liu R., Rong Y., Zhao M., Zhang J., Lai Y., Zhou Q., Wilz L.M., Li J., Vivona S. (2015). ATG14 promotes membrane tethering and fusion of autophagosomes to endolysosomes. Nat. Cell Biol..

[128] Sanchez-Wandelmer J., Reggiori F. (2013). Amphisomes: Out of the autophagosome shadow?. EMBO J..

[129] Cirone M. (2018). EBV and KSHV Infection Dysregulates Autophagy to Optimize Viral Replication, Prevent Immune Recognition and Promote Tumorigenesis. Viruses.

[130] English L., Chemali M., Duron J., Rondeau C., Laplante A., Gingras D., Desjardins M. (2009). Autophagy enhances the presentation of endogenous viral antigens on MHC class I molecules during HSV-1 infection. Nat. Immunol..

[131] Orvedahl A., Alexander D., Tallóczy Z., Sun Q., Wei Y., Zhang W. (2007). HSV-1 ICP34.5 confers neurovirulence by targeting the Beclin 1 autophagy protein. Cell Host. Microbe.

[132] Tallóczy Z., Jiang W., Virgin H.W., Leib D.A., Scheuner D., Kaufman R.J. (2002). Regula-tion of starvation- and virus-induced autophagy by the eIF2alpha kinase signaling pathway. Proc. Natl. Acad. Sci. USA.

[133] Tallóczy Z., Virgin H., Levine B. (2006). PKR-dependent autophagic degradation of herpes simplex virus type 1. Autophagy.

[134] Gobeil P.A., Leib D.A. (2012). Herpes simplex virus γ34.5 interferes with autophagosome maturation and antigen presentation in dendritic cells. mBio.

[135] Yakoub A.M., Shukla D. (2015). Basal Autophagy Is Required for Herpes simplex Virus-2 Infection. Sci. Rep..

[136] Lee H.K., Mattei L.M., Steinberg B.E., Alberts P., Lee Y.H., Chervonsky A., Mizushima N., Grinstein S., Iwasaki A. (2010). In Vivo Requirement for Atg5 in Antigen Presentation by Dendritic Cells. Immun..

[137] Lee D.Y., Sugden B. (2008). The LMP1 oncogene of EBV activates PERK and the unfolded protein response to drive its own synthesis. Blood.

[138] Takahashi M.N., Jackson W., Laird D.T., Culp T.D., Grose C., Haynes J.I. (2009). Varicella-zoster virus infection induces autophagy in both cultured cells and human skin vesicles. J. Virol..

[139] Graybill C., Morgan M.J., Levin M.J., Lee K.S. (2018). Varicella-zoster virus inhibits autophagosome-lysosome fusion and the degradation stage of mTOR-mediated autophagic flux. Virology.

[140] Buckingham E.M., Carpenter J.E., Jackson W., Grose C. (2014). Grose Autophagy and the effects of its inhibition on varicel-la-zoster virus glycoprotein biosynthesis and infectivity. J. Virol..

[141] Buckingham E.M., Girsch J., Jackson W., Cohen J.I., Grose C. (2018). Autophagy Quantification and STAT3 Expression in a Human Skin Organ Culture Model for Innate Immunity to Herpes Zoster. Front. Microbiol..

[142] Kenney S.C., Mertz J.E. (2014). Regulation of the latent-lytic switch in Epstein-Barr virus. Semin. Cancer Biol..

[143] Bhattacharjee S., Bose P., Patel K., Roy S.G., Gain C., Gowda H., Robertson E.S., Saha A. (2018). Transcriptional and epigenetic modulation of autophagy promotes EBV oncoprotein EBNA3C induced B-cell survival. Cell Death Dis..

[144] De Leo A., Colavita F., Ciccosanti F., Fimia G.M., Lieberman P.M., Mattia E. (2015). Inhibition of autophagy in EBV-positive Burkitt’s lymphoma cells enhances EBV lytic genes expression and replication. Cell Death Dis..

[145] Paludan C., Schmid D., Landthaler M., Vockerodt M., Kube D., Tuschl T., Münz C. (2005). Endogenous MHC class II processing of a viral nuclear antigen after autophagy. Science.

[146] Gilardini Montani M.S., Santarelli R., Granato M., Gonnella R., Torrisi M.R., Faggioni A., Cirone M. (2019). EBV reduces autophagy, intracellular ROS and mitochondria to impair monocyte survival and differentiation. Autophagy.

[147] Chaumorcel M., Lussignol M., Mouna L., Cavignac Y., Fahie K., Cotte-Laffitte J., Geballe A., Brune W., Beau I., Codogno P. (2011). The Human Cytomegalovirus Protein TRS1 Inhibits Autophagy via Its Interaction with Beclin 1. J. Virol..

[148] Taisne C., Lussignol M., Hernandez E., Moris A., Mouna L. (2019). Human cytomegalovirus hijacks the autophag-ic machinery and LC3 homologs in order to optimize cytoplasmic envelopment of mature infectious particles. Sci. Rep..

[149] Chaumorcel M., Souquère S., Pierron G., Codogno P., Esclatine A. (2008). Human cytomegalovirus controls a new autophagy-dependent cellular antiviral defense mechanism. Autophagy.

[150] Wen H.J., Yang Z., Zhou Y., Wood C. (2010). Enhancement of autophagy during lytic replication by the Kaposi’s sar-coma-associated herpesvirus replication and transcription activator. J. Virol..

[151] Pringle E.S., Robinson C.A., McCormick C. (2019). Kaposi’s Sarcoma-Associated Herpesvirus Lytic Replication Inter-feres with mTORC1 Regulation of Autophagy and Viral Protein Synthesis. J. Virol..

[152] Romeo M.A., Santarelli R., Montani M.S.G., Gonnella R., Benedetti R., Faggioni A., Cirone M. (2020). Viral Infection and Autophagy Dysregulation: The Case of HHV-6, EBV and KSHV. Cells.

[153] Lee J.-S., Li Q., Lee J.-Y., Lee S.-H., Jeong J.H., Lee H.-R., Chang H., Zhou F.-C., Gao S.-J., Liang C. (2009). FLIP-mediated autophagy regulation in cell death control. Nat. Cell Biol..

[154] Sinha S.C., Colbert C.L., Becker N., Wei Y., Levine B. (2008). Molecular basis of the regulation of Beclin 1-dependent autophagy by the gamma-herpesvirus 68 Bcl-2 homolog M11. Autophagy.

[155] Park S., Buck M.D., Desai C., Zhang X., Loginicheva E., Martinez J. (2016). Autophagy Genes Enhance Murine Gammaherpesvirus 68 Reactivation from Latency by Preventing Virus-Induced Systemic Inflamma-tion. Cell Host. Microbe.

[156] Leib D.A., Alexander D.E., Cox D., Yin J., Ferguson T.A. (2009). Interaction of ICP34.5 with Beclin 1 Modulates Herpes Simplex Virus Type 1 Pathogenesis through Control of CD4+ T-Cell Responses. J. Virol..

[157] Romeo M.A., Masuelli L., Gaeta A., Nazzari C., Granato M., Montani M.S.G., Faggioni A., Cirone M. (2019). Impact of HHV-6A and HHV-6B lytic infection on autophagy and endoplasmic reticulum stress. J. Gen. Virol..

[158] Edens B.M., Miller N., Ma Y.-C. (2016). Impaired Autophagy and Defective Mitochondrial Function: Converging Paths on the Road to Motor Neuron Degeneration. Front. Cell. Neurosci..

[159] Duarte L.F., Farías M.A., Álvarez D.M., Bueno S.M., Riedel C.A., González P.A. (2019). Herpes Simplex Virus Type 1 Infection of the Central Nervous System: Insights Into Proposed Interrelationships With Neurodegenerative Disorders. Front. Cell. Neurosci..

[160] Henne W.M., Buchkovich N.J., Emr S.D. (2011). The ESCRT pathway. Dev. Cell.

[161] Morita E. (2012). Differential requirements of mammalian ESCRTs in multivesicular body formation, virus budding and cell division. FEBS J..

[162] Schuh A.L., Audhya A. (2014). The ESCRT machinery: From the plasma membrane to endosomes and back again. Crit. Rev. Biochem. Mol. Biol..

[163] Hurley J.H., Hanson P.I. (2010). Membrane budding and scission by the ESCRT machinery: It’s all in the neck. Nat. Rev. Mol. Cell. Biol..

[164] Ibáñez F.J., Farías M.A., Gonzalez-Troncoso M.P., Corrales N., Duarte L.F., Retamal-Díaz A., González P.A. (2018). Experimental Dissection of the Lytic Replication Cycles of Herpes Simplex Viruses. Front. Microbiol..

[165] Hu Y.B., Dammer E.B., Ren R.J., Wang G. (2015). The endosomal-lysosomal system: From acidification and cargo sorting to neurodegeneration. Transl. Neurodegener.

[166] Wei B.L., Denton P.W., O’Neill E., Luo T., Foster J.L., Garcia J.V. (2005). Inhibition of lysosome and proteasome func-tion enhances human immunodeficiency virus type 1 infection. J. Virol..

[167] Spence J.S., He R., Hoffmann H.-H., Das T., Thinon E., Rice C.M., Peng T., Chandran K., Hang H.C. (2019). IFITM3 directly engages and shuttles incoming virus particles to lysosomes. Nat. Chem. Biol..

[168] Smith J.D., De Harven E. (1978). Herpes simplex virus and human cytomegalovirus replication in WI-38 cells. III. Cytochemical localization of lysosomal enzymes in infected cells. J. Virol..

[169] Kristen H., Sastre I., Muñoz-Galdeano T., Recuero M., Aldudo J., Bullido M.J. (2018). The lysosome system is severely impaired in a cellular model of neurodegeneration induced by HSV-1 and oxidative stress. Neurobiol. Aging.

[170] Finnen R.L., Mizokami K.R., Banfield B.W., Cai G.Y., Simpson S.A., Pizer L.I., Levin M.J. (2006). Postentry events are responsible for restriction of productive varicella-zoster virus infection in Chinese hamster ovary cells. J. Virol..

[171] Welsch S., Müller B., Kräusslich H.-G. (2007). More than one door—Budding of enveloped viruses through cellular membranes. FEBS Lett..

[172] Calistri A., Sette P., Salata C., Cancellotti E., Forghieri C., Comin A., Göttlinger H., Campadelli-Fiume G., Palù G., Parolin C. (2007). Intracellular trafficking and maturation of herpes simplex virus type 1 gB and virus egress require func-tional biogenesis of multivesicular bodies. J. Virol..

[173] Glosson N.L., Hudson A.W. (2007). Human herpesvirus-6A and -6B encode viral immunoevasins that downregulate class I MHC molecules. Virology.

[174] Brideau A., Enquist L., Tirabassi R. (2000). The role of virion membrane protein endocytosis in the herpesvirus life cycle. J. Clin. Virol..

[175] Mettenleiter T.C., Klupp B.G., Granzow H. (2006). Herpesvirus assembly: A tale of two membranes. Curr. Opin. Microbiol..

[176] Alconada A.U., Bauer B.S., Hoflack B. (1999). Intracellular traffic of herpes simplex virus glycoprotein gE: Char-acterization of the sorting signals required for its trans-Golgi network localization. J. Virol..

[177] Albecka A., Laine R.F., Janssen A.F., Kaminski C.F., Crump C.M. (2015). HSV-1 Glycoproteins Are Delivered to Virus Assembly Sites Through Dynamin-Dependent Endocytosis. Traffic.

[178] Hollinshead M., Johns H.L., Sayers C.L., Gonzalez-Lopez C., Smith G.L., Elliott G. (2012). Endocytic tubules regulated by Rab GTPases 5 and 11 are used for envelopment of herpes simplex virus. EMBO J..

[179] Bearer E.L., Wu C. (2019). Herpes Simplex Virus, Alzheimer’s Disease and a Possible Role for Rab GTPases. Front Cell Dev. Biol..

[180] Campadelli G., Brandimarti R., Di Lazzaro C., Ward P.L., Roizman B., Torrisi M.R. (1993). Fragmentation and dispersal of Golgi proteins and redistribution of glycoproteins and glycolipids processed through the Golgi apparatus after infection with herpes simplex virus 1. Proc. Natl. Acad. Sci. USA.

[181] Bagdonaite I., Norden R., Joshi H.J., Dabelsteen S., Nyström K., Vakhrushev S.Y., Olofsson S., Wandall H.H. (2015). A strategy for O-glycoproteomics of enveloped viruses--the O-glycoproteome of herpes simplex virus type 1. PLoS Pathog..

[182] Lebrun M., Lambert J., Riva L., Thelen N., Rambout X., Blondeau C. (2018). Varicella-Zoster Virus ORF9p Binding to Cellular Adaptor Protein Complex 1 Is Im-portant for Viral Infectivity. J. Virol..

[183] Das S., Vasanji A., Pellett P.E. (2007). Three-Dimensional Structure of the Human Cytomegalovirus Cytoplasmic Virion Assembly Complex Includes a Reoriented Secretory Apparatus. J. Virol..

[184] Alwine J.C. (2012). The Human Cytomegalovirus Assembly Compartment: A Masterpiece of Viral Manipulation of Cellular Processes That Facilitates Assembly and Egress. PLoS Pathog..

[185] Close W.L., Glassbrook J.E., Gurczynski S.J., Pellett P.E. (2018). Infection-Induced Changes Within the Endocytic Recycling Compartment Suggest a Roadmap of Human Cytomegalovirus Egress. Front. Microbiol..

[186] Torrisi M.R., Gentile M., Cardinali G., Cirone M., Zompetta C., Lotti L.V., Frati L., Faggioni A. (1999). Intracellular Transport and Maturation Pathway of Human Herpesvirus 6. Virology.

[187] Mori Y., Koike M., Moriishi E., Kawabata A., Tang H., Oyaizu H., Uchiyama Y., Yamanishi K. (2008). Human Herpesvirus-6 Induces MVB Formation, and Virus Egress Occurs by an Exosomal Release Pathway. Traffic.

[188] Pawliczek T., Crump C.M. (2009). Herpes Simplex Virus Type 1 Production Requires a Functional ESCRT-III Complex but Is Independent of TSG101 and ALIX Expression. J. Virol..

[189] Barnes J., Wilson D.W. (2019). Seeking Closure: How Do Herpesviruses Recruit the Cellular ESCRT Apparatus?. J. Virol..

[190] Hogue I.B., Scherer J., Enquist L.W. (2016). Enquist Exocytosis of Alphaherpesvirus Virions, Light Particles, and Glycoproteins Uses Constitutive Secretory Mechanisms. mBio.

[191] Lee C.-P., Chen M.-R. (2010). Escape of herpesviruses from the nucleus. Rev. Med. Virol..

[192] Reynolds A.E., Ryckman B.J., Baines J.D., Zhou Y., Liang L., Roller R.J. (2001). U(L)31 and U(L)34 proteins of herpes simplex virus type 1 form a complex that accumulates at the nuclear rim and is required for envelopment of nucleocapsids. J. Virol..

[193] Lv Y., Zhou S., Gao S., Deng H. (2019). Remodeling of host membranes during herpesvirus assembly and egress. Protein Cell.

[194] Turcotte S., Letellier J., Lippé R. (2005). Herpes Simplex Virus Type 1 Capsids Transit by the trans-Golgi Network, Where Viral Glycoproteins Accumulate Independently of Capsid Egress. J. Virol..

[195] Bjerke S.L., Roller R.J. (2006). Roles for herpes simplex virus type 1 UL34 and US3 proteins in disrupting the nuclear lamina during herpes simplex virus type 1 egress. Virology.

[196] Kato A., Kawaguchi Y. (2018). Us3 Protein Kinase Encoded by HSV: The Precise Function and Mechanism on Viral Life Cycle. Adv. Exp. Med. Biol..

[197] Saiz-Ros N., Czapiewski R., Epifano I., Stevenson A., Swanson S.K., Dixon C.R., Zamora D.B., McElwee M., Vijayakrishnan S., Richardson C.A. (2019). Host Vesicle Fusion Protein VAPB Contributes to the Nuclear Egress Stage of Herpes Simplex Virus Type-1 (HSV-1) Replication. Cells.

[198] Snyder A., Polcicova K., Johnson D.C. (2008). Herpes Simplex Virus gE/gI and US9 Proteins Promote Transport of both Capsids and Virion Glycoproteins in Neuronal Axons. J. Virol..

[199] Radtke K., Kieneke D., Wolfstein A., Michael K., Steffen W., Scholz T. (2010). Plus- and minus-end directed microtubule motors bind simultaneously to herpes simplex virus capsids using different inner tegument structures. PLoS Pathog..

[200] Foster T.P., Melancon J.M., Olivier T.L., Kousoulas K.G. (2004). Herpes simplex virus type 1 glycoprotein K and the UL20 protein are interdependent for intracellular trafficking and trans-Golgi network localization. J. Virol..

[201] Wang S., Mott K.R., Wawrowsky K., Kousoulas K.G., Luscher B., Ghiasi H. (2017). Binding of Herpes Simplex Virus 1 UL20 to GODZ (DHHC3) Affects Its Palmitoylation and Is Essential for Infectivity and Proper Targeting and Localization of UL20 and Glycoprotein K. J. Virol..

[202] Lau S.-Y.K., Crump C.M. (2015). HSV-1 gM and the gK/pUL20 Complex Are Important for the Localization of gD and gH/L to Viral Assembly Sites. Viruses.

[203] Naghavi M.H., Walsh D. (2017). Microtubule Regulation and Function during Virus Infection. J. Virol..

[204] Naghavi M.H., Gundersen G.G., Walsh D. (2013). Plus-end tracking proteins, CLASPs, and a viral Akt mimic regulate herpesvirus-induced stable microtubule formation and virus spread. Proc. Natl. Acad. Sci. USA.

[205] Brunetti C.R., Dingwell K.S., Wale C., Graham F.L., Johnson D.C. (1998). Herpes simplex virus gD and virions accu-mulate in endosomes by mannose 6-phosphate-dependent and -independent mechanisms. J. Virol..

[206] Temme S., Eis-Hübinger A.M., McLellan A.D., Koch N. (2010). The Herpes Simplex Virus-1 Encoded Glycoprotein B Diverts HLA-DR into the Exosome Pathway. J. Immunol..

[207] Niazy N., Temme S., Bocuk D., Giesen C., König A., Temme N. (2017). Misdirection of endosomal trafficking mediated by herpes simplex virus-encoded gly-coprotein B. FASEB J..

[208] Grabowska K., Wąchalska M., Graul M., Rychłowski M., Bieńkowska-Szewczyk K., Lipińska A.D. (2020). Alphaherpesvirus gB Homologs Are Targeted to Extracellular Vesicles, but They Differentially Affect MHC Class II Molecules. Viruses.

[209] Buckingham E.M., Jarosinski K.W., Jackson W., Carpenter J.E., Grose C. (2016). Exocytosis of Varicella-Zoster Virus Virions Involves a Convergence of Endosomal and Autophagy Pathways. J. Virol..

[210] Girsch J.H., Jackson W., Carpenter J.E., Moninger T.O., Jarosinski K.W., Grose C. (2020). Exocytosis of Progeny Infectious Varicella-Zoster Virus Particles via a Mannose-6-Phosphate Receptor Pathway without Xenophagy following Secondary Envelopment. J. Virol..

[211] Zerboni L., Sung P., Sommer M., Arvin A. (2018). The C-terminus of varicella-zoster virus glycoprotein M contains trafficking motifs that mediate skin virulence in the SCID-human model of VZV pathogenesis. Virology.

[212] Canitano A., Venturi G., Borghi M., Ammendolia M.G., Fais S. (2013). Exosomes released in vitro from Epstein–Barr virus (EBV)-infected cells contain EBV-encoded latent phase mRNAs. Cancer Lett..

[213] Zhao M., Nanbo A., Sun L., Lin Z. (2019). Extracellular Vesicles in Epstein-Barr Virus’ Life Cycle and Pathogenesis. Microorganisms.

[214] Hurwitz S.N., Nkosi D., Conlon M.M., York S.B., Liu X., Tremblay D.C., Meckes D.G. (2017). CD63 Regulates Epstein-Barr Virus LMP1 Exosomal Packaging, Enhancement of Vesicle Production, and Noncanonical NF-kappaB Signaling. J. Virol..

[215] Cepeda V., Esteban M., Fraile-Ramos A. (2010). Human cytomegalovirus final envelopment on membranes containing both trans-Golgi network and endosomal markers. Cell Microbiol..

[216] Das S., Pellett P.E. (2011). Spatial Relationships between Markers for Secretory and Endosomal Machinery in Human Cytomegalovirus-Infected Cells versus Those in Uninfected Cells. J. Virol..

